# Underdiagnosis of Major Depressive Episodes in Hemodialysis Patients: The Need for Screening and Patient Education

**DOI:** 10.3390/jcm10184109

**Published:** 2021-09-11

**Authors:** Wojciech Orzechowski, Wiktor Buczek, Joanna Emma Szczerba, Ryszard Gellert, Andrzej Rydzewski, Bartosz Fiderkiewicz, Paweł Żebrowski, Dorota Daniewska, Andrzej Kokoszka

**Affiliations:** 1 Psychiatry Day Ward, Mazowiecki Brodnowski Hospital, 03-242 Warsaw, Poland; wojciech.orzechowski@wum.edu.pl; 2 II Department of Psychiatry, Medical University of Warsaw, 02-091 Warsaw, Poland; wiktor.buczek@wum.edu.pl; 3 Psychiatry Ward, Mazowiecki Brodnowski Hospital, 03-242 Warsaw, Poland; j.szczerba@brodnowski.pl; 4 Department of Internal Medicine, Nephrology and Transplantology, Centre of Postgraduate Medical Education, 01-809 Warsaw, Poland; ryszard.gellert@cmkp.edu.pl (R.G.); dorotadm@wp.pl (D.D.); 5 Department of Internal Medicine, Nephrology and Transplantology, Central Clinical Hospital of the Ministry of Interior and Administration, 02-507 Warsaw, Poland; andrzej.rydzewski@cmkp.edu.pl (A.R.); abfider@interia.pl (B.F.); 6 Department of Nephrology, Dialysis, and Internal Medicine, Medical University of Warsaw, 02-097 Warsaw, Poland; pzebrowski@wum.edu.pl

**Keywords:** hemodialysis, depression, major depressive episode, MDE, major depressive disorder, MDD, nonadherence, compliance, conformance, treatment refusal

## Abstract

This article aims to identify the reasons why patients with major depressive episode (MDE) do not seek treatment for their mental disorder. 89 out of 208 persons screened were diagnosed with major depressive episode using the Mini-International Neuropsychiatric Interview. 85 individuals with untreated depression filled out the following questionnaires: Beck Depression Inventory, List of Explanations of Well-Being (LEWB), Brief Measure to Assess Perception of Self-Influence on the Course of the Disease, Coping Inventory for Stressful Situations, Brief Method of Evaluating Coping with Disease, and Metacognitions Questionnaire. There were 43 women (50.6%) and 42 men (49.4%), aged 24 to 93 years (Mean (M) = 68.26 years; Standard Deviation (SD) = 14.19 years), with dialysis vintage ranging from 1 month to 33 years (M = 70.63 months; SD = 75.26 months). Among study patients, 70.6% declared that depression was the cause of their poor well-being, 75.3% attributed their depressive symptoms to kidney failure, and 49.4%, more specifically, to hemodialysis. A total of 64.7% of patients had a low perception of self-influence on the course of their kidney disease, and 58.5% presented a coping style focused on emotions. The most frequent dysfunctional metacognitive beliefs were negative beliefs about not controlling one’s own thoughts. This attitude was related to the low perception of self-influence on the course of the disease, maladaptive coping styles, and dysfunctional metacognitive beliefs.

## 1. Introduction

Major depressive disorder (MDD) is a seriously disabling disease that in 2007 became the third leading cause of years of healthy life lost due to disability (YLDs) in all age groups [1]. Its prevalence rate is much higher in women than in men [2]. According to a meta-analysis, the prevalence of depression at the last stage of kidney failure ranged from 22.8% to 39.3% in studies in which the diagnosis was established based on clinical history taking [3]. In a recent study by Kokoszka et al. [4], depressive disorders were also diagnosed with the use of the structured Mini-International Neuropsychiatric Interview (MINI) 5.0.0 [5,6] in 78.5% of 84 hemodialysis patients, including 29.0% with major depressive episode (MDE). The prevalence of depression in hemodialysis patients is higher (52%) than in those with other chronic diseases (42%) and those without chronic diseases (10%) [7]. Depression in patients receiving dialysis leads to increased mortality [8], longer hospitalization time [9], longer hemodialysis vintage, and less frequent transplants [10]. A total of 39% of hemodialyzed patients with MDD lived two years up to the follow-up, whereas the survival ratio for those without an MDD was 95% [10]. Data have shown that among hemodialysis patients suffering from depression, 57% seek help [11]. Sixteen to forty-five percent indeed receive professional help in this matter [12], and over 70% feel reluctant to starting treatment [13]. Nearly 70% of patients undergoing dialysis who had depression were not aware that the symptoms they experienced were indeed those of depression and, at the same time, they did not think that they needed help [13]. According to our best knowledge, there have been no publications exploring the reasons why many hemodialysis patients with depressive symptoms do not report them to their physicians.

When looking for potential reasons for not seeking medical attention by the patients who experience low mood, or outright marked depressive symptoms, factors related to nonadherence to diabetes management can be considered as a model [14]. Research findings have also indicated the importance of self-influence on the course of the disease and coping mechanisms. Moreover, evidence emphasizes the role of metacognitive processes in depression and its therapy. Thus, it can be inferred that metacognitive processes may also influence patients’ attitudes toward depressive symptoms.

In chronic, progressive diseases, the concept of exerting control [15] over their course is not entirely useful in understanding of their management as they may worsen with time, thus making complete control impossible. A more adequate notion here is the perception of one’s self-influence on the disease course, which was defined as the extent of belief about one’s own ability to shape the course of the disease. It has been formulated [9] that the coping style adopted in response to a particular stressor depends on the perceived degree of control over that stressor. Even in terminal states, when control of disease progression is impossible, a person can influence the course of the disease to some degree. Self-influence also differs from perceived self-efficacy, which is defined as the belief in one’s ability to produce certain levels of action that influence events affecting one’s life. More specifically, self-efficacy beliefs determine how people feel, think, behave, and motivate themselves [16,17], whereas the perception of self-influence is related to disease management and is therefore more precise. Perception of self-influence on the disease course was found to be a significant predictor of engagement in treatment for type 2 diabetes [14] and schizophrenia [18,19].

At least some sense of influence over the problem causing the stress response is necessary for coping that problem. This defines coping styles. There may be styles oriented on performing the task [20,21] or aimed at finding the best solution [14,22]. Lacking a perception of one’s own impact on the stressor means that only avoidance or emotional coping styles can be employed.

The metacognitive theory of psychologic disorders [23] suggests that the way a person thinks about their thinking (metacognitive beliefs) affects their psychologic well-being. This has been confirmed in a wide range of psychologic disorders [24,25] and suggests that metacognitive beliefs become cognitive vulnerability factors of psychologic distress in general. Dysfunctional metacognitive beliefs were found among patients with depression [26]. According to our best knowledge, there are no data on metacognitive beliefs in patients receiving dialysis who have MDD.

The main aim of this study was to identify reasons why patients with MDE do not seek treatment for psychiatric symptoms. An additional goal was to verify the hypothesis that the patient’s perception of their self-influence on the course of the disease and their coping styles are meaningful factors affecting their attitude toward depressive symptoms in the course of hemodialysis treatment.

## 2. Materials and Methods

### 2.1. The Pilot Study—General Remarks

The pilot study aimed at the assessment of obstacles encountered in the treatment of depression in hemodialysis patients. A total of 123 hemodialysis patients participated in the pilot study in Warsaw; 23 individuals (18.47%) from this group withdrew from the study. Depressive symptoms were found in 100 patients (59 men and 41 women), 30 of whom met the criteria of MDD according to MINI. In 30 patients, symptoms of subclinical depression were recognized based on the Hamilton Depression Rating Scale and Beck Depression Inventory.

Patients’ age ranged from 32 to 92 years (M = 65.92 years, SD = 14.59 years), and dialysis vintage, from 1 to 282 months (i.e., up to 23.5 years) (M = 46.45 months, SD = 52.87 months). There were no significant differences in terms of age, dialysis time, or sex ratio between the groups of patients with MDD and subclinical depression. None of the dialysis patients with MDD had sought psychiatric or psychologic treatment, while among those suffering from subclinical depression, only 2 individuals had benefited from this type of treatment.

The List of Explanations of Well-Being (LEWB) for hemodialysis patients was constructed in order to carry out this study (File S1). The prevailing conviction of patients with diagnosed MDD and a statistically comparable number of those with subclinical depression (86%) was that their current health status was related to their well-being, i.e., how they were feeling. Moreover, patients with MDD tended to consider kidney disease (70%) and dialysis (73%) as factors responsible for their well-being. Patients with subclinical depression shared the view that kidney disease affected their well-being in 57% of cases, and the influence of dialysis was emphasized in 50% of this population. At the same time, 67% of patients with MDD and 86% of those with subclinical depression did not identify potential problems related to their well-being as symptoms of kidney failure. Among patients diagnosed with depression, 77% did not think that their current well-being qualifies as symptoms of depression, and none of patients with subclinical depression considered depression to be the cause of their well-being. Since starting dialysis, as many as 80% of patients diagnosed with MDD had no opportunity to discuss their mental well-being with hospital staff, and 70% had no contact with a psychologist or psychiatrist.

The results indicated that among patients diagnosed with MDD, the absence of any professional psychologic support in their immediate environment was the dominant view. This prompted us to perform another study of a larger patient population in order to elucidate and confirm initial findings from the pilot study.

### 2.2. The Main Study

#### 2.2.1. Study Design and Patient Characteristics

A total of 246 consecutive patients with chronic kidney disease who were undergoing hemodialysis were identified and invited to participate in the study in the following medical centers: (1) Department of Nephrology and Internal Medicine, Centre for Postgraduate Medical Education, Bielanski Hospital, (2) Department of Nephrology, Dialysis, and Internal Medicine, Medical University of Warsaw, Central Teaching Clinical Hospital, and (3) Department of Internal Medicine, Nephrology and Transplantology, Central Clinical Hospital of the Ministry of Interior and Administration.

Thirty-eight (15.47%) of the 246 persons invited refused to take part in the study: 11 (4.47%) provided no reason for this decision, and 27 (11.0%) stated that their somatic condition (pain, fatigue, disability) was too severe to join the study group. Those who did sign an informed consent form were screened for MDE using the “Major Depressive Episode” module from the structured clinical interview MINI 5.0.0 [6].

Eighty-nine (42.8%) patients with MDE were identified; only four of them (4.5%) had received treatment for MDE. The core study included 85 persons with MDD who had not started antidepressive treatment before our investigations. There were 42 males and 43 females, aged between 24 and 93 years (M = 68.26 years, SD = 14.19 years). Patients had been on hemodialysis for 1 month to 33 years (M = 70.63 months; SD = 75.26 months). The following comorbidities were diagnosed by MINI 5.0.0: hypomanic episode, N = 1 (1.2%); panic disorder, N = 5 (5.9%); panic disorder with agoraphobia, N = 7 (8.2%); agoraphobia, N = 5 (5.9%); social phobia, N = 6 (7.1%); obsessive-compulsive disorder, N = 3 (3.5%); and acute stress disorder, N = 1 (1.2%). The study population was predominantly at low risk of suicidal behavior, which was revealed based on responses of 88.2% of the respondents.

**Figure 1:**
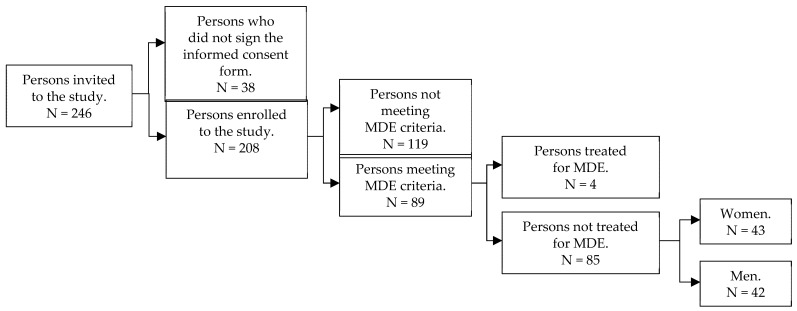
Study flowchart.

The study flowchart (Figure 1) shows groups of patients identified at subsequent stages of the main study. Detailed demographic and clinical characteristics of hemodialysis patients with untreated MDE included in the study are presented in Table 1.

#### 2.2.2. Methods

All patients who signed informed consent were screened for MDE using the Major Depressive Episode module of MINI 5.0.0. All who met MDE criteria while screened, were interviewed using the full version of MINI 5.0.0, filled out a set of questionnaires. All methods and questionnaires used in this study are briefly described below:

1. The Mini-International Neuropsychiatric Interview (MINI) version 5.0.0 based on Diagnostic and Statistical Manual of Mental Disorders, Fourth Edition (DSM-IV) and International Classification of Diseases, Tenth Revision (ICD-10) criteria [6]. Originally, this instrument was developed as a short, structured interview to diagnose mental disorders based on the Diagnostic and Statistical Manual of Mental Disorders, Third Edition—Revised (DSM-III-R) and ICD-10 criteria. It can be used by clinicians after a short training, although nonprofessionals need a more intensive course. The psychometric properties of the original, English-language version of MINI were assessed as very high on the basis of Composite International Diagnostic Interview (CIDI) [6]. Considering the diagnosis of depression, sensitivity and specificity of the tool were 94% and 79%, respectively, and the kappa coefficient was 0.83.
2. The Beck Depression Inventory, a tool used to assess the severity of depression. It is a self-completion questionnaire with a total score ranging from 0 to 84 points, with scores described as normal (≤9), indicating mild depression (10–18), indicating moderate depression (19–29), and indicating severe depression (≥30) [27,28]. The reliability assessment showed internal consistency—as measured by a Cronbach alpha coefficient of 0.86 for psychiatric patients and 0.81 for nonpsychiatric patients—and high agreement with clinical assessments on the Hamilton Depression Rating Scale (HDRS) [29]. BDI was used in the study for a purpose that is not the essence of this work, therefore, its results are not presented.
3. The List of Explanations of Well-Being (LEWB) for persons with undiagnosed and untreated depression among patients with chronic kidney disease undergoing hemodialysis. The first version of this tool was developed for the pilot study described above. Its items were identified based on preliminary discussions with nephrologists and group interviews with dialysis patients, who were asked to determine the possible causes of their depressive symptoms and the potential obstacles to discussing these issues with medical staff. Due to the length and complexity of the original version, the newer version was modified for the current study and only statements that respondents most often referred to during the pilot study were retained. Patient responses can be classified in three categories, which means that depressive disorders may be caused by: (1) somatic diseases, (2) mental disorders, and (3) factors other than illness. The tool is a self-rating list. Patients are asked questions about their beliefs regarding the cause of their well-being. Respondents mark their answers on a 5-point Likert scale, and the predefined answers are as follows: I (1) strongly disagree, (2) would rather disagree, (3) have no opinion, (4) would rather agree, or (5) strongly agree. The questionnaire can be found in the supplementary files, see File S1.
4. The Brief Measure to Assess Perception of Self-Influence on the Course of the Disease, version for hemodialysis patients [20]—a self-assessment scale consisting of 11 items to which the patient responds using a 5-point Likert scale. The reliability of the scale as measured by Cronbach alpha coefficient is 0.9, and accuracy as measured by Kendall tau coefficient is 0.6. The scale, although brief, is characterized by very high reliability and satisfactory accuracy. It assigns patients to one of the three groups according to their perception of self-influence on the course of kidney disease, i.e., (1) high (score ≤ 1.1), (2) moderate (1.1 < score ≤ 2.1), or (3) low (score > 2.1) perception of self-influence on the course of the disease.
5. The Brief Method of Evaluating Coping with Disease, with versions for men and for women [30]. This is a tool used to determine the dominant style the person uses to cope with disease. The scale of 4 items tailored to the interests of both women and men corresponds with 4 different styles oriented on (1) a task, (2) searching for the best solution, (3) emotions, and (4) avoidance. The questionnaire is characterized by good reliability:
  (a) Cronbach alpha is 0.71 for females and 0.75 for males—for the style focused on a solution.
  (b) Cronbach alpha is 0.55 for females and 0.59 for males—for the style focused on searching for the best solution.
  (c) Cronbach alpha is 0.67 for females and 0.68 for males—for the style focused on emotions.
  (d) Cronbach alpha is 0.65 for females and 0.67 for males—for the style focused on avoidance.

This tool is characterized by a moderate validity of the scales, as measured by the correlation of individual scales with the Coping Inventory for Stressful Situations (CISS) questionnaire, i.e., r = 0.42 for task-oriented coping and r = 0.29 for emotion-oriented coping.

6. The Coping Inventory for Stressful Situations (CISS) [31]—a tool used to diagnose styles of coping with stress. The results are presented on three scales: (1) focused on a task, (2) focused on emotions, and (3) focused on avoidance. It includes 2 forms of behavior: (a) engaging in surrogate activities and (b) seeking social contact. The questionnaire is characterized by high internal consistency of individual scales (Cronbach alpha coefficients within the range of 0.78–0.90) and satisfactory stability (correlation coefficients between tests at intervals of 2–3 weeks in the range of 0.73–0.80). The tool presents factor validity. It was also tested for theoretical and criterion validity (here by comparing the CISS results of various professional and clinical groups).
7. The Metacognitions Questionnaire (MCQ) [32]—a tool that aims to examine metacognitive beliefs. The scale consists of 65 statements that create five separate dimensions within which the respondent chooses answers on a 4-point Likert-type scale. The investigated dimensions are: (1) positive beliefs on worrying (e.g., “Worrying helps me avoid problems in the future”) (Cronbach alpha = 0.87); (2) negative beliefs about not controlling one’s own thoughts, and danger (e.g., “If I let my worrying thoughts get out of control, they will end up controlling me”) (Cronbach alpha = 0.89); (3) beliefs about cognitive certainty (e.g., “I have a poor memory”) (Cronbach alpha = 0.84); (4) general negative beliefs, including responsibility, punishment, and superstition (e.g., “It is bad to think certain thoughts”) (Cronbach alpha = 0.74); and (5) cognitive self-awareness (e.g., “I monitor my thoughts”) (Cronbach alpha = 0.72).

#### 2.2.3. Statistical Analysis

The following statistical tests were used to analyze study data: the Mann-Whitney U test [33], ANOVA 2-way analysis of variance [34], Pearson chi-square test of independence [35], Spearman rho correlation [36], and multiple correspondence analysis [37]. For all tests, statistical significance was set at *p* = 0.05.

## 3. Results

### 3.1. Well-Being

Patients’ responses to LEWB (Table 2) indicated that the vast majority of those with undiagnosed and untreated MDD had not taken into consideration the fact that they were suffering from a mental disorder. According to the results of the questionnaire (responses are presented in Table 2), 75.3% of the study participants attributed their poor well-being just to kidney disease.

Nearly 90% of those patients perceived their well-being as related to their medical conditions, i.e., current health state, and 60% of patients believed that their poor well-being was due to causes other than the disease, e.g., family problems, unemployment, etc. The least frequently indicated causes of poor well-being were the fact of being dialyzed (49%) and having kidney failure (42%).

A total of 70.6% of the patients who described their poor well-being as a symptom of depression did not consider it to be a mental disorder, but rather a poor mood. It is worth noting, however, that about half of the patients definitely did not associate their depressive state with chronic kidney disease or dialysis treatment.

### 3.2. Perception of Self-Influence on the Course of Kidney Disease

Based on patients’ responses to The Brief Measure to Assess Perception of Self-Influence on the Course of the Disease, version for hemodialysis patients (Table 3), the mean level of perception of self-influence on the disease course among study participants was low (M = 2.35, SD = 83, Median = 2.50), according to the Polish reference values [38]:

- A mean score ≤1.1 was a threshold for a high perception of self-influence on disease progression.
- A mean score 1.1 < X ≤ 2.1 marked a moderate perception of self-influence on disease progression.
- A mean score >2.1 indicated a low perception of self-influence on disease progression.

Over half of the respondents (N = 55; 64.7%) showed a low perception of self-influence on disease progression. Only 9 persons (12.9%) had a high perception of self-influence on disease progression.

### 3.3. Styles of Coping with the Disease and Stressful Situations

According to the Brief Method of Evaluating Coping with Disease, the largest group of patients (N = 36; 42.4%) was diagnosed with an emotion-oriented coping passivity (Table 4). The least numerous group (N = 9; 10.6%) consisted of those with the best solution–oriented coping style.

There were statistically significant (chi-square = 9.913; *p* = 0.019) differences between men and women undergoing hemodialysis in the adaptive styles of coping with disease. Women were most often (N = 24; 28.2%) characterized by emotion-oriented coping, whereas men most commonly (N = 18; 21.2%) showed avoidance-oriented coping.

Nevertheless, according to the CISS results (Table 5), the largest group (N = 48; 58.5%) included hemodialysis patients characterized by emotion-related coping with stressful situations, and avoidance-oriented coping was identified least frequently (N = 3; 3.7%). 

### 3.4. Metacognitive Beliefs

The evaluation of metacognitive beliefs as measured by MCQ showed that hemodialysis patients with untreated depression most often experienced negative beliefs about not controlling their own thoughts and danger (M = 41.54, P = 65%) (Table 6).

## 4. Discussion

Looking for the answer to the question “Why are MDEs often underdiagnosed and left untreated in hemodialysis patients?”, we performed analyses focused on patients’ explanations for their poor well-being and potential reasons for not reporting depressive symptoms to physicians. Unfortunately, the presented data showed that doctors did not ask patients carefully enough about the symptoms of depression. Therefore—most likely for this reason—so many cases of depression are not diagnosed in dialysis patients. The investigator—a psychologist who used the MDD module of MINI 5.0.0.—could identify individuals with this disorder. Of note, screening for MDD by asking two questions is very effective. A positive answer to one of the following questions: (1) Did you often feel depressed or hopeless during the last month? and (2) Did you often lack interest in undertaking various activities or a feeling of pleasure during these activities? has a sensitivity of 97% and a specificity of 67% for the diagnosis of MDD [39]. Readily available tools—the Patient Health Questionnaire 9 (PHQ-9) [40,41,42] and the World Health Organization-Five Well-Being Index (WHO-5)—are also short and effective screening methods for depression [43,44,45].

Awareness of the presence of subclinical depressive disorders among both dialysis patients and their physicians is of great importance. A recently published study [46] showed a high percentage of hemodialysis patients affected by undetected syndromes such as irritability identified by the Diagnostic Criteria for Psychosomatic Research (DCPR) [47]. This report is congruent with data from a pilot study, which found that the prevalence of depression among hemodialysis patients was 30%. It also seems significant that 30% of dialysis patients—due to diagnosed subclinical depression—can be classified as a potential “group at risk”. The two groups do not differ significantly in terms of age, sex ratio, or dialysis vintage. Patients also agreed that their mood was intrinsically associated with their kidney disease and at the same time did not consider their mood to be a symptom of it. Instead, patients significantly differed with regard to the association of their well-being with dialysis therapy (73% of patients with MDD and 50% of patients with subclinical depression) and in classifying their current well-being as a symptom of depression (77% of patients with MDD; none of the patients with subclinical depression shared this view). It also correlated with twice as many patients with MDD (60%) being willing to see a mental-health professional compared with patients with subclinical depression (30%).

The relatively low participation refusal rate (15.5%) and use of the structured MINI are the strong points of this study. The application of the MINI module for the diagnosis of MDD during screening, conducted by a trained interviewer, could have been the reason why MDD was diagnosed in 42.8% of the study group, which is an approximately 2-fold higher percentage than found in the results of a meta-analysis of studies using self-rating scales, which was 22.8% [3]. The lack of a control group of persons undergoing hemodialysis in whom MDD was recognized and treated is indeed a disadvantage of this study but, considering that this applied for only 4.5% of all patients with MDD, it would be necessary to enlarge the group of screened patients to approximately 1500, which was beyond the extent of this study. Therefore, further studies including larger samples and control groups are needed to confirm our results.

Our findings are congruent with other data indicating that resistance to treatment of depression was present in 70% of dialysis patients [13]. We showed that the majority (85.9%) of patients perceived their poor well-being status as related to their current medical state and did not consider themselves as persons with a mental disorder. This emphasizes the need to make dialysis patients aware of the risks of MDD and of the differences among this disorder, depressive symptoms of adjustment disorder, subclinical depression, and natural sadness. Although 60% of the group reported having depression, it did not mean that they had MDD in mind. In the context of other responses to LEWB, it is more likely that they reported experiencing depressive symptoms understood as a natural reaction to their current medical condition.

This is in line with the low rate of task-oriented coping (20.7%) and problem solving–oriented coping styles (including best solution–oriented coping) (25.9%). Maladaptive coping and coping styles focused on avoidance, and reduction of stress-related emotions, are probably associated with the lack of proper management of experienced MDD symptoms.

The low level of awareness of self-influence on disease progression is also in line with the described findings and indicates the direction of therapeutic interventions. They should include making patients aware of the possibilities of MDD treatment with medication and psychotherapy. This could enhance patients’ perception of self-influence on the course of the disease, and eventually improve their coping with both MDD and renal failure. Also, challenging their dysfunctional metacognitive negative beliefs about not controlling their own thoughts and danger, could be helpful as part of those interventions.

## 5. Conclusions

MDD is often unrecognized by the treating physicians of dialysis patients. The high prevalence of MDD in this population implies the need to routinely use a screening tool to recognize MDD. The vast majority of hemodialysis patients with MDD do not believe that their distress is caused by a mental disorder. This indicates the need to educate patients about the risks of MDD and to determine, whether they are aware of the differences between low mood and MDD. Many patients need help to improve their stress management, including increasing their perception of self-influence on the disease course. Negative metacognitive beliefs, particularly regarding the patient’s failure to control own thoughts, should be recognized and, if present, challenged in the process of coping with MDD symptoms.

## Figures and Tables

**Table 1:** Demographic and clinical characteristics of dialysis patients with untreated MDE (N = 85).

Variables		Values
Age		68.26 ^1^ (14.19) ^2^ years old
Dialysis duration		70.63 ^1^ (75.26) ^2^ months
**Total**		**N (%)**
Attitude to treatment of depression (N = 89)	Treated	4 (4.5%)
	Untreated	85 (95.5%)
Patient’s sex	Female	43 (50.6%)
	Male	42 (49.4%)
Patient’s education level	Primary school	13 (19.7%)
	Vocational	13 (19.7%)
	Secondary school	25 (37.9%)
	High	15 (22.7%)
Time of hemodialysis	Morning	43 (50.6%)
	Afternoon	22 (25.9%)
	Evening	20 (23.5%)
Hemodialysis center	Dpt. of Nephrology, Dialysis, and Int. Medicine, Medical Univ. of Warsaw, Central Teaching Hospital	24 (28.2%)
	Dpt. of Int. Medicine, Nephrology and Transplantology, Central Clinical Hospital of the Ministry of Interior and Administration	29 (34.1%)
	Dpt. of Nephrology and Int. Medicine, Centre for Postgraduate Medical Education, Bielanski Hospital	32 (37.6%)
Risk of suicidal behavior	Low	75 (88.2%)
	Moderate	10 (11.8%)
Current diagnosed comorbidities	Hypomanic episode	1 (1.2%)
	Panic disorder	5 (5.9%)
	Panic disorder with agoraphobia	7 (8.2%)
	Agoraphobia	5 (5.9%)
	Social phobia	6 (7.1%)
	Obsessive-compulsive disorder	3 (3.5%)
	Acute stress disorder	1 (1.2%)

^1^ Mean (M); ^2^ Standard Deviation (SD).

**Table 2:** Well-being of patients with untreated depression according to the List of Explanations of Well-Being (LEWB), aggregated responses (detailed data in File S2).

My Well-Being Is:	Agree ^1^		Disagree ^2^	
	N	%	N	%
A symptom of kidney failure	36	42.3	43	50.6
Affected by hemodialysis	42	49.4	39	45.9
Related to my other diseases and/or conditions	47	55.3	37	43.5
Related to factors other than illness, e.g., family problems, unemployment, etc.	52	61.2	31	36.5
Inherently related to a disease like mine (kidney disease)	64	75.3	18	21.2
A symptom of depression	60	70.6	16	18.8
Not related to my current health state	8	7.2	76	89.4

^1^ “strongly agree” and “would rather agree” answers only; ^2^ “strongly disagree” and “would rather disagree” answers only.

**Table 3:** Perception of self-influence on the disease course among patients with untreated MDD in the setting of kidney disease.

Low	Moderate	High
N = 55	N = 19	N = 11
64.7%	22.4%	12.9%

**Table 4:** Styles of coping with the disease (based on the Brief Method of Evaluating Coping with Disease).

Task-Oriented Coping		Best Solution-Oriented Coping		Emotion-Oriented Coping		Avoidance-Oriented Coping	
N = 13		N = 9		N = 36		N = 27	
15.3%		10.6%		42.4%		31.8%	
**F ^1^**	**M ^2^**	**F**	**M**	**F**	**M**	**F**	**M**
N = 4	N = 9	N = 6	N = 3	N = 24	N = 12	N = 9	N = 18
4.7%	10.6%	7.1%	3.5%	28.2%	14.1%	10.6%	21.2%

^1^ F—Female; ^2^ M—Male.

**Table 5:** Styles of coping with stressful situations (based on CISS).

Task-Oriented	Emotion-Oriented	Avoidance-Oriented	Two Styles More Apparent than the Third Style
N = 17	N = 48	N = 3	N = 14
20.7%	58.5%	3.7%	17.1%

**Table 6:** Metacognitive beliefs: descriptive statistics for the study sample.

Beliefs	M ^1^	P ^2^	SD ^3^	Median
Positive worry beliefs	26.95	35%	10.23	23.00
Negative beliefs about not controlling one’s own thoughts and danger	41.54	65%	12.97	43.00
Beliefs about cognitive confidence	19.25	48%	8.44	17.00
General negative beliefs about thoughts, including a sense of responsibility, superstition, and expectation of punishment	23.51	45%	5.20	25.00
Beliefs about cognitive self-awareness	15.84	57%	8.09	13.00

^1^ M = Mean; ^2^ P = Percentage of the maximum score; ^3^ SD = Standard deviation.

## Data Availability

The raw data are in possession of Wojciech Orzechowski.

## Supplementary Materials

The following are available online at www.mdpi.com/article/10.3390/jcm10184109/s1, File S1: List of Explanations of Well-Being (LEWB), File S2: Well-being of patients with untreated depression according to the List of Explanations of Well-Being (LEWB).
