# Supplementary material for: Underdiagnosis of Major Depressive Episodes in Hemodialysis Patients: The Need for Screening and Patient Education
Source: J Clin Med. 2021 Sep 11;10(18):4109. doi: 10.3390/jcm10184109 (PMC8465545; doi:10.3390/jcm10184109)
Supplement: Supplementary file 1 [file jcm-10-04109-s001.zip › File S1.pdf]

The List of Explanations of Well-Being (LEWB)

The following statements are about the patient’s mental well-being.

Please mark one answer for each statement:

1. **My well-being is inherently related to a disease like mine (renal failure).**

Regarding the above statement I:

a. strongly disagree b. rather disagree c. have no opinion d. rather agree e. strongly agree

2. **My well-being is related to my other diseases and/or conditions.**

Regarding the above statement I:

a. strongly disagree b. rather disagree c. have no opinion d. rather agree e. strongly agree

3. **My well-being is not related to my current health condition.**

Regarding the above statement I:

a. strongly disagree b. rather disagree c. have no opinion d. rather agree e. strongly agree

4. **My well-being is a symptom of renal failure.**

Regarding the above statement I:

a. strongly disagree b. rather disagree c. have no opinion d. rather agree e. strongly agree

5. **My well-being is due to dialysis.**

Regarding the above statement I:

a. strongly disagree b. rather disagree c. have no opinion d. rather agree e. strongly agree

6. **My well-being is a symptom of depression.**

Regarding the above statement I:

a. strongly disagree b. rather disagree c. have no opinion d. rather agree e. strongly agree

7. **My well-being is related to factors other than illness, e.g. family problems, lack of work, etc.**

Regarding the above statement I:

a. strongly disagree b. rather disagree c. have no opinion d. rather agree e. strongly agree
