# Supplementary material for: Underdiagnosis of Major Depressive Episodes in Hemodialysis Patients: The Need for Screening and Patient Education
Source: J Clin Med. 2021 Sep 11;10(18):4109. doi: 10.3390/jcm10184109 (PMC8465545; doi:10.3390/jcm10184109)
Supplement: Supplementary file 1 [file jcm-10-04109-s001.zip › File S2.pdf]

Well-being of patients with untreated depression according to the List of Explanations of Well-Being (LEWB), aggregated responses – detailed data.

**My well-being is:**	**I strongly disagree**	**Rather disagree**	**I have no opinion**	**Rather agree**	**I strongly agree**
inherently related to a disease like mine (renal failure)	8.2% N=7	12.9% N=11	3.5% N=3	21.2% N=18	54.1% N=46
related to my other diseases and/or conditions	40.0% N=34	3.5% N=3	1.2% N=1	3.5% N=3	51.8% N=44
is not related to my current health condition	85.9% N=73	3.5% N=3	2.4% N=2	7,1% N=6	1.2% N=1
a symptom of renal failure	36.5% N=31	14.1% N=12	7.1% N=6	12.9% N=11	29.4% N=25
due to hemodialysis	35.3% N=30	10.6% N=9	4.7% N=4	12.9% N=11	36.5% N=31
a symptom of depression	4.7% N=4	14.1% N=12	10.6% N=9	11.8% N=10	58.8% N=50
related to factors other than illness, e.g. family problems, lack of work, etc.	34.1% N=29	2.4% N=2	2.4% N=2	5.9% N=5	55.3% N=47
